# Risk factors and development of machine learning diagnostic models for lateral lymph node metastasis in rectal cancer: multicentre study

**DOI:** 10.1093/bjsopen/zrae073

**Published:** 2024-07-17

**Authors:** Shunsuke Kasai, Akio Shiomi, Hideyuki Shimizu, Monami Aoba, Yusuke Kinugasa, Takuya Miura, Kay Uehara, Jun Watanabe, Kazushige Kawai, Yoichi Ajioka

**Affiliations:** Division of Colon and Rectal Surgery, Shizuoka Cancer Center, Shizuoka, Japan; Department of Gastrointestinal Surgery, Tokyo Medical and Dental University, Tokyo, Japan; Division of Colon and Rectal Surgery, Shizuoka Cancer Center, Shizuoka, Japan; Department of AI Systems Medicine, M&D Data Science Center, Tokyo Medical and Dental University, Tokyo, Japan; Department of AI Systems Medicine, M&D Data Science Center, Tokyo Medical and Dental University, Tokyo, Japan; Department of Gastrointestinal Surgery, Tokyo Medical and Dental University, Tokyo, Japan; Department of Gastroenterological Surgery, Hirosaki University, Graduate School of Medicine, Aomori, Japan; Department of Gastrointestinal and Hepato-Biliary-Pancreatic Surgery, Nippon Medical School Hospital, Tokyo, Japan; Department of Surgery, Gastroenterological Center, Yokohama City University Medical Center, Yokohama, Japan; Department of Surgery, Tokyo Metropolitan Cancer and Infectious Diseases Center, Komagome Hospital, Tokyo, Japan; Division of Molecular and Diagnostic Pathology, Graduate School of Medical and Dental Sciences, Niigata University, Niigata, Japan

## Abstract

**Background:**

The diagnostic criteria for lateral lymph node metastasis in rectal cancer have not been established. This research aimed to investigate the risk factors for lateral lymph node metastasis and develop machine learning models combining these risk factors to improve the diagnostic performance of standard imaging.

**Method:**

This multicentre prospective study included patients who underwent lateral lymph node dissection without preoperative treatment for rectal cancer between 2017 and 2019 in 15 Japanese institutions. First, preoperative clinicopathological factors and magnetic resonance imaging findings were evaluated using multivariable analyses for their correlation with lateral lymph node metastasis. Next, machine learning diagnostic models for lateral lymph node metastasis were developed combining these risk factors. The models were tested in a training set and in an internal validation cohort and their diagnostic performance was tested using receiver operating characteristic curve analyses.

**Results:**

Of 212 rectal cancers, 122 patients were selected, including 232 lateral pelvic sides, 30 sides of which had pathological lateral lymph node metastasis. Multivariable analysis revealed that poorly differentiated/mucinous adenocarcinoma, extramural vascular invasion, tumour deposit and a short-axis diameter of lateral lymph node ≥ 6.0 mm were independent risk factors for lateral lymph node metastasis. Patients were randomly divided into a training cohort (139 sides) and a test cohort (93 sides) and machine learning models were computed on the basis of a combination of significant features (including: histological type, extramural vascular invasion, tumour deposit, short- and long-axis diameter of lateral lymph node, body mass index, serum carcinoembryonic antigen level, cT, cN, cM, irregular border and mixed signal intensity). The top three models with the highest sensitivity in the training cohort were as follows: support vector machine (sensitivity, 1.000; specificity, 0.773), light gradient boosting machine (sensitivity, 0.950; specificity, 0.918) and ensemble learning (sensitivity, 0.950; specificity, 0.917). The diagnostic performances of these models in the test cohort were as follows: support vector machine (sensitivity, 0.750; specificity, 0.667), light gradient boosting machine (sensitivity, 0.500; specificity, 0.852) and ensemble learning (sensitivity, 0.667; specificity, 0.864).

**Conclusion:**

Machine learning models combining multiple risk factors can contribute to improving diagnostic performance of lateral lymph node metastasis.

## Teaser

The diagnostic criteria for lateral lymph node metastasis in rectal cancer have not been established. This multicentre study in Japan revealed that poorly differentiated or mucinous adenocarcinoma, extramural vascular invasion, tumour deposit and short-axis diameter of lateral lymph node ≥ 6.0 mm were independent risk factors for lateral lymph node metastasis. Machine learning models combining these multiple risk factors contribute to improving diagnostic performance of lateral lymph node metastasis.

## Introduction

Surgery is the standard treatment for rectal cancer. Since William Ernest Miles first examined the lymphatic spread of rectal cancer in detail^1^, surgical treatment for reliable local control of the deep and narrow pelvis has been sought. In Japan, surgery alone has been the principal treatment, even for locally advanced low rectal cancer, and total mesorectal excision (TME) plus lateral lymph node dissection (LLND) has been promoted as a reliable surgical technique for local control in the central and lateral pelves^2^. In particular, some retrospective studies have shown that LLND is highly effective for rectal cancer with lateral lymph node metastasis (LLNM)^3,4^. However, performing LLND is sometimes technically demanding, and there are inevitable concerns about complications, such as prolonged operating time, increased blood loss and increased urogenital dysfunction^5,6^. Therefore, in Western countries, TME combined with preoperative chemoradiotherapy (CRT) without LLND is the standard treatment for locally advanced low rectal cancer^7,8^.

However, in recent years, there have been an increasing number of studies on insufficient local control after TME plus preoperative CRT for rectal cancer with enlarged lateral lymph nodes that are suspected to be LLNM^9^. The safe and effective technique of LLND established in Japan has been gradually spreading worldwide^10,11^, and there has been a growing international momentum that LLND should be performed for rectal cancer with preoperative suspicion of LLNM^12–14^.

Although magnetic resonance imaging (MRI) is the standard modality for the preoperative diagnosis of LLNM by assessing the size and morphology of lymph nodes^15,16^, the diagnostic criteria for LLNM have not been established and in the Japanese guidelines the criteria for omitting LLND are unclear^2^. In addition to the imaging assessment of lymph nodes, clinicopathological risk factors for LLNM have been examined, and some studies have attempted to combine these risk factors to improve the diagnostic performance of LLNM^17–19^. However, these attempts have shown limited diagnostic performance and have not been applied in daily practice.

Improving diagnostic performance is essential in various medical fields, and the use of artificial intelligence (AI) has attracted attention in recent years^20,21^. Combining multiple factors to obtain a single answer is a specialty of machine learning, which is a subfield of AI, and an attempt at a single institution was made to use machine learning to diagnose LLNM^19^. In that study, although the machine learning diagnostic model for LLNM was significantly more useful than the conventional diagnostic method using the short-axis diameter of the lateral lymph nodes, the need for further studies was emphasized to examine novel risk factors related to LLNM and develop better machine learning models. Thus, the present study aims first to investigate the true risk factors for LLNM using data from several Japanese institutions on the diagnostic performance of high-resolution MRI for rectal cancer, and second, to develop a machine learning model that combines these risk factors to improve the diagnostic performance of LLNM.

## Methods

### Patients

This study prospectively registered patients who underwent TME plus LLND for primary rectal adenocarcinoma between January 2017 and December 2019 at 15 institutions in the Japanese Society for Cancer of the Colon and Rectum (JSCCR) MRI Study Group, independently from clinical stages. Patients were excluded if they received preoperative treatment, if they did not undergo preoperative MRI and if they had concomitant cancers. Patients were evaluated if they underwent unilateral or bilateral LLND. Of note, the eligible patients were analysed on each lateral pelvic side: a patient with bilateral LLND was deemed as having two sides, and one with unilateral LLND was deemed as having one side. All study protocols were approved by the Ethics Committee of the University of Tokyo Hospital (No. 11406-[5]), and all patients provided written informed consent.

### MRI assessment and treatment strategy for locally advanced low rectal cancers

Patients underwent high-resolution T2-weighted MRI with a 3 mm slice thickness before surgery. The short- and long-axis diameters of all lateral lymph nodes were measured, and their morphology, such as irregular border and mixed signal intensity, was assessed without clinical information at each institution. In addition to evaluating rectal cancer based on the Japanese tumour-node-metastasis classification^22^, extramural vascular invasion (EMVI) and tumour deposit (TD) were evaluated using MRI by two colorectal surgeons with the assistance of a radiologist without any clinical information. Positive EMVI was defined as an MRI-EMVI score of 3 or 4^23^, and TD was defined as an irregular nodule within the mesorectum that directly interrupted the course of veins but was discontinuous from the primary tumour^24^. Treatment strategy for locally advanced low rectal cancer was at the discretion of the participating centres. Based on the Japanese guidelines, the indication for LLND is a low rectal cancer, which is defined as a tumour located distal to the peritoneal reflection, staged as cT3–4 anyN or cT1–2 rectal cancer with LLNM on preoperative images^2^. However, LLND was sometimes omitted, or preoperative treatment was added according to each institution’s criteria. Although administration of preoperative chemotherapy and/or CRT was allowed, patients who received preoperative treatment were excluded from this study. All open, laparoscopic and robotic TME procedures including LLND were considered eligible, and LLND required at least unilateral systematic dissection of both the internal iliac and obturator nodes.

### Risk factors and development of machine learning diagnostic models for LLNM

First, using the data set of lateral pelvic sides included, a logistic regression analysis was performed to examine the risk factors for LLNM based on preoperative clinicopathological factors and MRI findings. The clinicopathological factors included the age, sex, body mass index (BMI), serum carcinoembryonic antigen (CEA) level, carbohydrate antigen 19-9 level, histological type, cT, cN and cM. In addition, the MRI findings included EMVI, TD, the morphology of lateral lymph node (irregular border and mixed signal intensity) and the size of the lateral lymph node (short- and long-axis diameter). The cutoff values for the short- and long-axis diameter to diagnose LLNM were established using the receiver operating characteristic (ROC) curve analyses.

Variables with *P* values < 0.05 in the univariable analysis were included in the multivariable model. Next, the data set was divided into the training and an internal validation test set in a 6:4 ratio using random stratified sampling, using a Python library ‘scikit-learn’, specifically the ‘train_test_split’ method in ‘sklearn.model_selection’. To create the machine learning models, the following two methods were used to select statistically significant combinations of features based on features documented by the multivariable logistic regression analysis. The first method used the Python module ‘statsmodels’, which explored and provided statistical data of each explanatory variable in the logistic regression. Using this module in the training set, *P* values for the coefficients of the independent features in each combination were found. From all the combinations, only those features with a *P* < 0.05 were selected. The second method used the univariable logistic regression analysis to select features with *P* < 0.05. The combinations of features identified by the above two methods were computed into eight types of algorithms and created machine learning models. The algorithms used were five single classifiers (logistic regression, light gradient boosting machine (LightGBM), extreme gradient boosting (XGBoost), random forest (RF), and support vector machine (SVM)) and three ensemble classifiers (logistic regression + LightGBM + SVM, logistic regression +XGBoost + SVM and logistic regression + RF + SVM), using the Python library ‘scikit-learn’ (class ‘Logistic Regression’ for logistic regression, ‘Random Forest Classifier’ for RF, ‘SVC’ for SVM, and ‘Base Estimator’ and ‘Classifier Mixin’ for ensemble classifiers), and the Python packages ‘LightGBM’ and ‘XGBoost’.

Before or after selecting the more sensitive feature combinations, we tuned the hyperparameters of the machine learning models. The evaluation metric for hyperparameter optimization was sensitivity evaluated by stratified five-fold validation. The Python library ‘Optuna’ was used for optimization, and the ‘StratifiedKFold’ method in ‘sklearn.model_selection’ for stratified five-fold validation. The optimization details for each algorithm are listed in *Supplementary material*, *Table S1*. Each machine learning model was evaluated based on its sensitivity to the training set, calculated by a stratified five-fold validation. For the top three models with the highest sensitivity in the training cohort, the ROC curves, area under the ROC curve (AUC), sensitivity, specificity and SHapley Addictive exPlanations (SHAP) values in the test cohort were calculated.

### Statistical analyses

Fisher’s exact test was used to assess categorical variables, and the Mann–Whitney *U* test was used to compare continuous variables between the groups. Two-sided *P* values < 0.05 were considered statistically significant. All statistical analyses, other than machine learning, were performed using EZR (Saitama Medical Center, Jichi Medical University, Saitama, Japan)^25^.

## Results

**Fig. 1:**
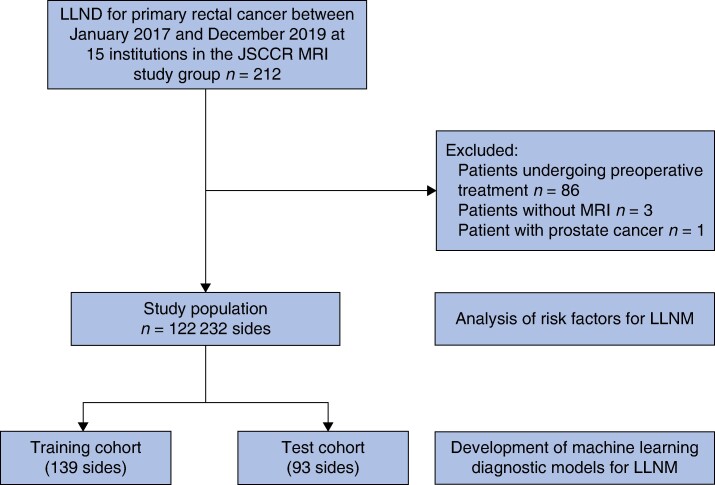
Patient selection LLND, lateral lymph node dissection; JSCCR, Japanese Society for Cancer of the Colon and Rectum; MRI, magnetic resonance imaging; LLNM, lateral lymph node metastasis.

Of 212 patients with rectal cancer eligible and treated during the study interval, 122 patients were evaluated, including 12 who underwent unilateral LLND and 110 who underwent bilateral LLND. Overall, 232 lateral pelvic sides were included: 30 sides were positive for pathological LLNM (LLNM+) and 202 sides without pathological LLNM (LLNM–) (*Fig. 1*). *Table 1* compares the characteristics between the LLNM+ and LLNM− groups. The two groups showed significant differences in CEA level, the histological type, cT and cN. Additionally, EMVI (90.0% *versus* 58.9%, *P* < 0.001) and TD (40.0% *versus* 11.9%, *P* < 0.001) were significantly higher in the LLNM+ group than in the LLNM− group. The MRI findings of the lateral lymph nodes showed the LLNM+ group had a significantly more irregular border (60.0% *versus* 14.4%, *P* < 0.001) and mixed signal intensity (56.7% *versus* 10.4%, *P* < 0.001) and significantly larger short-axis diameter (7.7 mm *versus* 4.0 mm, *P* < 0.001) than the LLNM− group. *Table 2* presents the risk factors for LLNM. Multivariable analysis revealed that histological type (poorly differentiated or mucinous adenocarcinoma), EMVI, TD and short-axis diameter of lateral lymph node ≥ 6.0 mm were independent risk factors for LLNM. The distribution of the sides with LLNM according to risk factors using preoperative MRI is shown in *Supplementary material*, *Fig. S1*; in addition, the ROC curves of the short- and long-axis diameter of lateral lymph node for diagnosing LLNM are shown in *Supplementary material*, *Fig. S2*.

**Table 1:** Patient characteristics

	LLNM+ (30 sides)	LLNM− (202 sides)	*P*
Age (years), median (range)	62 (35–82)	64 (35–82)	0.700
**Sex**			
Male	20 (66.7)	129 (63.9)	0.840
Female	10 (33.3)	73 (36.1)	
BMI (kg/m^2^), median (range)	22.0 (17.4–32.9)	22.4 (15.4–34.0)	0.676
CEA (ng/ml), median (range)	6.2 (1.0–106.4)	3.8 (0.5–452.6)	0.039
CA19-9 (U/ml), median (range)	16 (2–169)	11 (2–545)	0.148
Tumour distance from the anal verge (cm), median (range)	4.0 (0–11.0)	5.0 (0–11.0)	0.159
**Histological type**			
Papillary/well differentiated/moderately differentiated	24 (80.0)	196 (97.0)	0.002
Poorly differentiated/mucinous	6 (20.0)	6 (3.0)	
**cT**			
1	0 (0)	0 (0)	0.037
2	3 (10.0)	12 (5.9)	
3	17 (56.7)	157 (77.7)	
4	10 (33.3)	33 (16.3)	
**cN**			
0	2 (6.7)	71 (35.1)	< 0.001
1	9 (30.0)	50 (24.8)	
2	5 (16.7)	48 (23.8)	
3	14 (46.7)	33 (16.3)	
**cM**			
0	26 (86.7)	184 (91.1)	0.500
1	4 (13.3)	18 (8.9)	
EMVI on MRI	27 (90.0)	119 (58.9)	< 0.001
TD on MRI	12 (40.0)	24 (11.9)	< 0.001
Irregular border on MRI	18 (60.0)	29 (14.4)	< 0.001
Mixed signal intensity on MRI	17 (56.7)	21 (10.4)	< 0.001
Long-axis diameter of LLN (mm) on MRI, median (range)	9.3 (2.8–28.2)	6.9 (1.8–44.4)	0.206
Short-axis diameter of LLN (mm) on MRI, median (range)	7.7 (1.5–22.5)	4.0 (1.3–8.8)	< 0.001
**pT, median (range)**			
1	1 (3.3)	6 (3.0)	0.003
2	1 (3.3)	49 (24.3)	
3	19 (63.3)	126 (62.4)	
4	9 (30.0)	21 (10.4)	
**pN, median (range)**			
0	0 (0)	116 (57.4)	< 0.001
1	0 (0)	60 (29.7)	
2	0 (0)	26 (12.9)	
3	30 (100)	0 (0)	

Values are n (%) unless otherwise indicated. LLNM+, lateral lymph node metastasis; LLNM−, no lateral lymph node metastasis; BMI, body mass index; CEA, carcinoembryonic antigen; CA19-9, carbohydrate antigen 19-9; EMVI, extramural vascular invasion; MRI, magnetic resonance imaging; TD, tumour deposit; LLN, lateral lymph node.

**Table 2:** Results of univariable and multivariable analyses of risk factors for lateral lymph node metastasis

	Univariable analysis		Multivariable analysis	
	OR (95% c.i.)	*P*	OR (95% c.i.)	*P*
Age ≥ 65 (*versus* < 65) (years)	1.020 (0.474, 2.200)	0.960		
Sex: male (*versus* female)	1.130 (0.503, 2.550)	0.765		
BMI ≥ 22 (*versus* < 22) (kg/m^2^)	0.888 (0.412, 1.910)	0.761		
CEA ≥ 5.1 (*versus* < 5.1) (ng/ml)	2.650 (1.210, 5.810)	0.015	0.871 (0.249, 3.040)	0.829
CA19–9 ≥ 38 (*versus* < 38) (U/ml)	1.740 (0.688, 4.430)	0.241		
Histological type poorly/mucinous (*versus* papillary/well differentiated/moderately differentiated)	8.170 (2.440, 27.300)	< 0.001	53.700 (3.980, 724.00)	0.003
cT ≥ 4 (*versus* cT ≤ 3)	2.560 (1.100, 5.970)	0.029	1.200 (0.370, 3.880)	0.762
cN ≥ 1 (*versus* cN0)	7.590 (1.760, 32.800)	0.007	4.020 (0.314, 51.600)	0.285
cM ≥ 1 (*versus* cM0)	1.570 (0.494, 5.010)	0.444		
EMVI (*versus* absence)	6.280 (1.840, 21.400)	0.003	16.800 (1.460, 195.000)	0.024
Tumour deposit (*versus* absence)	4.940 (2.120, 11.500)	< 0.001	3.730 (1.020, 13.600)	0.047
Irregular border (*versus* absence)	8.950 (3.900, 20.500)	< 0.001	2.510 (0.748, 8.400)	0.136
Mixed signal intensity (*versus* absence)	11.300 (4.810, 26.400)	< 0.001	1.310 (0.359, 4.790)	0.682
Long-axis diameter of LLN ≥ 8.0 (*versus* < 8.0) (mm)	4.190 (1.780, 9.880)	0.001	0.446 (0.094, 2.120)	0.310
Short-axis diameter of LLN ≥ 6.0 (*versus* < 6.0) (mm)	19.500 (7.830, 48.400)	< 0.001	17.000 (3.090, 94.000)	< 0.001

OR, odds ratio; BMI, body mass index; CEA, carcinoembryonic antigen; CA19-9, carbohydrate antigen 19-9; EMVI, extramural vascular invasion; LLN, lateral lymph node.

To develop the machine learning models, selected patients were divided into a training cohort (139 sides) and a test cohort (93 sides). There were no significant differences in any of these features between the two cohorts (*Supplementary material*, *Table S2*). The following two methods were tested to select the statistically significant feature combinations. First, using the Python module ‘statsmodels’ in the training cohort, two combinations of features including the histological type (poorly differentiated or mucinous adenocarcinoma), EMVI, TD and short-axis diameter of lateral lymph node ≥ 6.0 mm, were tested and only the combination of features with a *P* value of <0.05 were selected. One of the combinations selected included the four features previously mentioned (histological type, EMVI, TD and short-axis diameter of lateral lymph node) and BMI; the other combination selected included the four features plus BMI and cM. Second, 64(=2^6) combinations of the four features (histological type, EMVI, TD and short-axis diameter of lateral lymph node) and the other six features with *P* values < 0.05 at the univariable analysis (CEA ≥ 5.1 ng/ml, cT ≥ 4, cN ≥ 1, irregular border, mixed signal intensity, long-axis diameter of lateral lymph node ≥ 8.0 mm) were tested. Thus, the authors input the 66 combinations identified by the above two methods into eight types of machine learning algorithms and created 528 machine learning models.

**Fig. 2:**
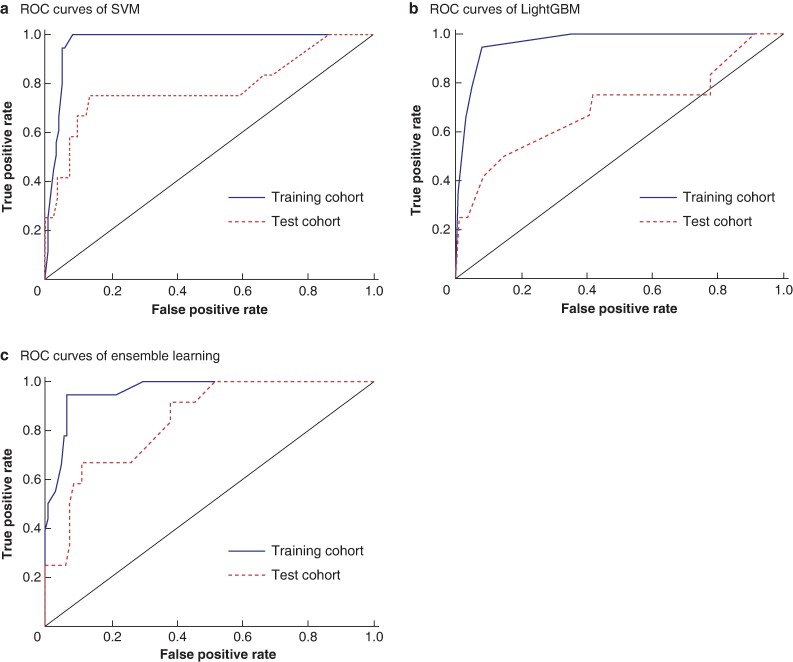
Receiver operating characteristic curves of machine learning diagnostic models for lateral lymph node metastasis ROC, receiver operating characteristic; SVM, support vector machine; LightGBM, light gradient boosting machine; ensemble learning, logistic regression + LightGBM +SVM.

*Table 3* shows the diagnostic performance of the top three models with the highest sensitivity in the training cohort. The model with the highest sensitivity in the training cohort was SVM with inputs of the four features, CEA ≥ 5.1 ng/ml, cN ≥ 1, irregular border and mixed signal intensity. The second highest model was LightGBM with inputs of the four features and CEA ≥ 5.1 ng/ml. The third highest model was ensemble learning (logistic regression + LightGBM + SVM) with inputs of the four essential features, CEA ≥ 5.1 ng/ml, cN ≥ 1, mixed signal intensity and long-axis diameter of lateral lymph node ≥ 8.0 mm. *Figure 2* presents the ROC curves of the three models for the training and test cohorts. The AUC for SVM in the training cohort was 0.970 and in the test cohort was 0.781. The AUC for LightGBM in the training cohort was 0.964 and in the test cohort was 0.692. The AUC for ensemble learning in the training cohort was 0.962 and in the test cohort was 0.840. The SHAP values for each model are shown in *Supplementary material*, *Figs S3–S5*. For SVM, an irregular border was considered most important for the diagnosis of LLNM, and for the other two machine learning models, a short-axis diameter of lateral lymph node ≥ 6.0 mm was considered most important.

**Table 3:** Diagnostic performance of machine learning models for lateral lymph node metastasis

Machine learning model	SVM	LightGBM	Ensemble learning
**Training cohort**			
AUC	0.970	0.964	0.962
Sensitivity	1.000	0.950	0.950
Specificity	0.773	0.918	0.917
PPV	0.414	0.656	0.665
NPV	1.000	0.990	0.990
**Test cohort**			
AUC	0.781	0.692	0.840
Sensitivity	0.750	0.500	0.667
Specificity	0.667	0.852	0.864
PPV	0.250	0.333	0.421
NPV	0.947	0.920	0.946

SVM, support vector machine; LightGBM, light gradient boosting machine; ensemble learning, logistic regression + LightGBM + SVM; AUC, area under the receiver operating characteristic curve; PPV, positive predictive value; NPV, negative predictive value.

## Discussion

This study investigated the risk factors for LLNM using a multicentre data set in Japan. Preoperative clinicopathological factors and high-resolution MRI findings identified the risk factors for LLNM, including: the histological type (poorly differentiated or mucinous adenocarcinoma), EMVI, TD and short-axis diameter of lateral lymph node ≥ 6.0 mm. The use of highly sensitive machine learning models has the potential to improve the diagnostic performance of LLNM diagnosis.

Measurement of the diameter of the lateral lymph nodes using MRI is considered the most useful method for diagnosis of LLNM. However, there is no consensus on whether to measure the short- or long-axis diameter of the lateral lymph nodes, and each study has proposed various cutoff values^12,26,27^. The main analysis of the JSCCR MRI Study Group evaluated in detail the relationship between the diameter of the lateral lymph nodes and LLNM and suggested omitting criteria for LLND^28^. In this study, ROC curve analysis set a cutoff value for the short-axis diameter of lateral lymph nodes as ≥ 6.0 mm and identified it as a significant risk factor for LLNM. Although MRI findings of irregular border and mixed signal intensity in enlarged lymph nodes could improve the diagnostic performance of LLNM^16^, it was difficult not to miss LLNM below the defined cutoff value.

In recent years, EMVI and TD, identified using MRI, have attracted attention as factors that strongly influence the prognosis of patients with rectal cancer^24^. A recent study in this field suggested that these factors could lead to distant metastasis via the ‘anatomic highway’^29^. The present study also suggested that these factors could lead to LLNM via the ‘anatomic highway’. Just as distant metastasis spreads from EMVI through the inferior mesenteric vein, LLNM can spread through the lymphatic vessels, which are accompanied by the middle rectal artery^30^. Although several retrospective studies in Japan have reported that EMVI is a risk factor for LLNM^31,32^, the present study also found similar results in a larger number of patients in multiple institutions. Considering that TD is caused by the development of EMVI^29^, it is reasonable to identify EMVI and TD as risk factors for LLNM.

Although several risk factors have been investigated to improve the diagnostic performance of LLNM^33^, how these risk factors are combined to diagnose LLNM remains controversial. One study attempted to examine the prediction of LLNM using a logistic model that included risk factors such as preoperative lateral lymph node status, histopathological grade and pathological perirectal lymph node status^17^. Additionally, another study identified a combination of the short-axis diameter of lateral lymph node, tumour location, EMVI and short-axis diameter of the perirectal lymph node as optimal for the prediction of LLNM and developed a nomogram using these factors^18^. As well as the conventional statistical combination of multiple risk factors to improve the diagnostic performance of LLNM, machine learning, a novel AI, has recently been suggested to be effective^19^. These attempts have developed LLNM prediction models that are more useful than conventional diagnostic methods using the size of the lateral lymph node alone, but are insufficient to diagnose patients, before surgery, with LLNM who should truly undergo LLND.

In this study, three highly sensitive machine learning diagnostic models for LLNM were developed to combine multiple preoperative risk factors to have better diagnostic performance than what has been previously reported^17–19^. In addition, considering that the prevalence of LLNM in rectal cancer was relatively rare and that LLND should be performed for patients with LLNM^3,4^, it was significant that these diagnostic models had high negative predictive values and could, before surgery, identify patients who should undergo LLND. These diagnostic models always included the four features (histological type (poorly differentiated or mucinous adenocarcinoma), EMVI, TD and short-axis diameter of lateral lymph node ≥ 6.0 mm), which were identified as risk factors for LLNM. Given the distribution of sides with LLNM according to risk factors, it is important to assess EMVI in addition to the short-axis diameter of the lateral lymph node to avoid missing patients with LLNM. Furthermore, the SHAP values of each machine learning model showed features considered important for LLNM diagnosis. However, the machine learning models developed in this study are not universal, and the field of AI is ever-evolving. Additionally, novel methods for LLNM diagnosis, such as molecular profiling and AI-based imaging, are being investigated^34,35^. Therefore, machine learning that combines multiple risk factors for LLNM is considered a useful method, and models should be constantly improved.

The present study has some limitations. First, the sample size was relatively small. Although this was a well-designed multicentre study using high-resolution MRI, more patients were required to develop more effective machine learning models, because patients with LLNM were rare. To demonstrate the true effectiveness of the models developed in this study, these models need to be validated in other cohorts. Second, the authors analysed the eligible patients on each side. In the case of bilateral LLND, information on patient characteristics, including the rectal tumour and mesorectum, was the same on each side. In addition, the presence or absence of LLNM on each side, rather than individual lymph nodes, was evaluated. Thus, the detailed evaluation of the presence or absence of individual lymph node metastases required careful analysis of each patient and lymph node. Third, patients who received preoperative treatment were excluded because the standard treatment strategy for rectal cancer in Japan is upfront surgery^2^, and imaging evaluation after preoperative treatment is relatively complicated^36^. Although the need for local control by LLND has been recognized in Western countries, the main concern is how and in whom LLND should be performed, especially after preoperative treatment^37^. To solve this problem, it is necessary to develop machine learning models for the diagnosis of LLNM after preoperative treatment by adding various features before and after preoperative treatment. However, it is important to consider appropriate treatment strategies for locally advanced low rectal cancer with LLNM and discuss whether preoperative treatment is really necessary. Therefore, a reliable method for diagnosing LLNM before preoperative treatment should be developed first.

In conclusion, the risk factors for LLNM were histological type (poorly differentiated or mucinous adenocarcinoma), EMVI, TD and short-axis diameter of lateral lymph node ≥ 6.0 mm. In the diagnosis of LLNM using high-resolution MRI, it is useful to measure the size of the lateral lymph nodes and assess EMVI and TD. Additionally, the authors developed highly sensitive machine learning diagnostic models for LLNM by combining these risk factors. Since an accurate preoperative diagnosis of LLNM is crucial to perform highly effective LLND for locally advanced low rectal cancer, further studies must continue to analyse novel risk factors for LLNM and develop more accurate diagnostic methods.

## Supplementary Material

zrae073_Supplementary_Data

## Data Availability

The data sets generated and analysed during the present study are available from the corresponding author upon reasonable request.
