# Supplementary material for: Risk factors and development of machine learning diagnostic models for lateral lymph node metastasis in rectal cancer: multicentre study
Source: BJS Open. 2024 Jul 17;8(4):zrae073. doi: 10.1093/bjsopen/zrae073 (PMC11252850; doi:10.1093/bjsopen/zrae073)
Supplement: zrae073_Supplementary_Data [file zrae073_supplementary_data.zip › Supplementary_Materials.docx]

**Risk factors and development of machine learning diagnostic models for lateral lymph node metastasis in rectal cancer: a multicentre study**

Shunsuke Kasai^1,2^, Akio Shiomi^1^, Hideyuki Shimizu^3^, Monami Aoba^3^, Yusuke Kinugasa^2^, Takuya Miura^4^, Kay Uehara^5^, Jun Watanabe^6^, Kazushige Kawai^7^, Yoichi Ajioka^8^

1Division of Colon and Rectal Surgery, Shizuoka Cancer Center, Shizuoka, Japan

2Department of Gastrointestinal Surgery, Tokyo Medical and Dental University, Tokyo, Japan

3Department of AI Systems Medicine, M＆D Data Science Center, Tokyo Medical and Dental University, Tokyo, Japan

4Department of Gastroenterological Surgery, Hirosaki University, Graduate School of Medicine, Aomori, Japan

5Department of Gastrointestinal and Hepato-Biliary-Pancreatic Surgery, Nippon Medical School Hospital, Tokyo, Japan

6Department of Surgery, Gastroenterological Center, Yokohama City University Medical Center, Yokohama Japan

7Department of Surgery, Tokyo Metropolitan Cancer and Infectious Diseases Center, Komagome Hospital, Tokyo, Japan

8Division of Molecular and Diagnostic Pathology, Graduate School of Medical and Dental Sciences, Niigata University, Niigata, Japan

**Corresponding author:** Akio Shiomi, Division of Colon and Rectal Surgery, Shizuoka Cancer Center, 1007 Shimonagakubo, Nagaizumi-cho, Sunto-gun, Shizuoka 411-8777, Japan, Tel.: +81-55-989-5222, Fax: +81-55-989-5551, Email: [a.shiomi@scchr.jp](mailto:a.shiomi@scchr.jp)

**Supplementary Materials - Index**

| **Supplementary Figures and Tables** |  |
| --- | --- |
| Figure S1 | *pag. 3* |
| Figure S2 | *pag. 4* |
| Figure S3 | *pag. 5* |
| Figure S4 | *pag. 6* |
| Figure S5 | *pag. 7* |
| Table S1 | *pag. 8* |
| Table S2 | *pag. 9-10* |

**Supplementary Figures and Tables**

**Figure S1** Distribution of sides with lateral lymph node metastasis according to the risk factors using preoperative magnetic resonance imaging

LLNM +, sides with pathological lateral lymph node metastasis; EMVI, extramural vascular invasion; LLN, lateral lymph node; and TD, tumour deposit


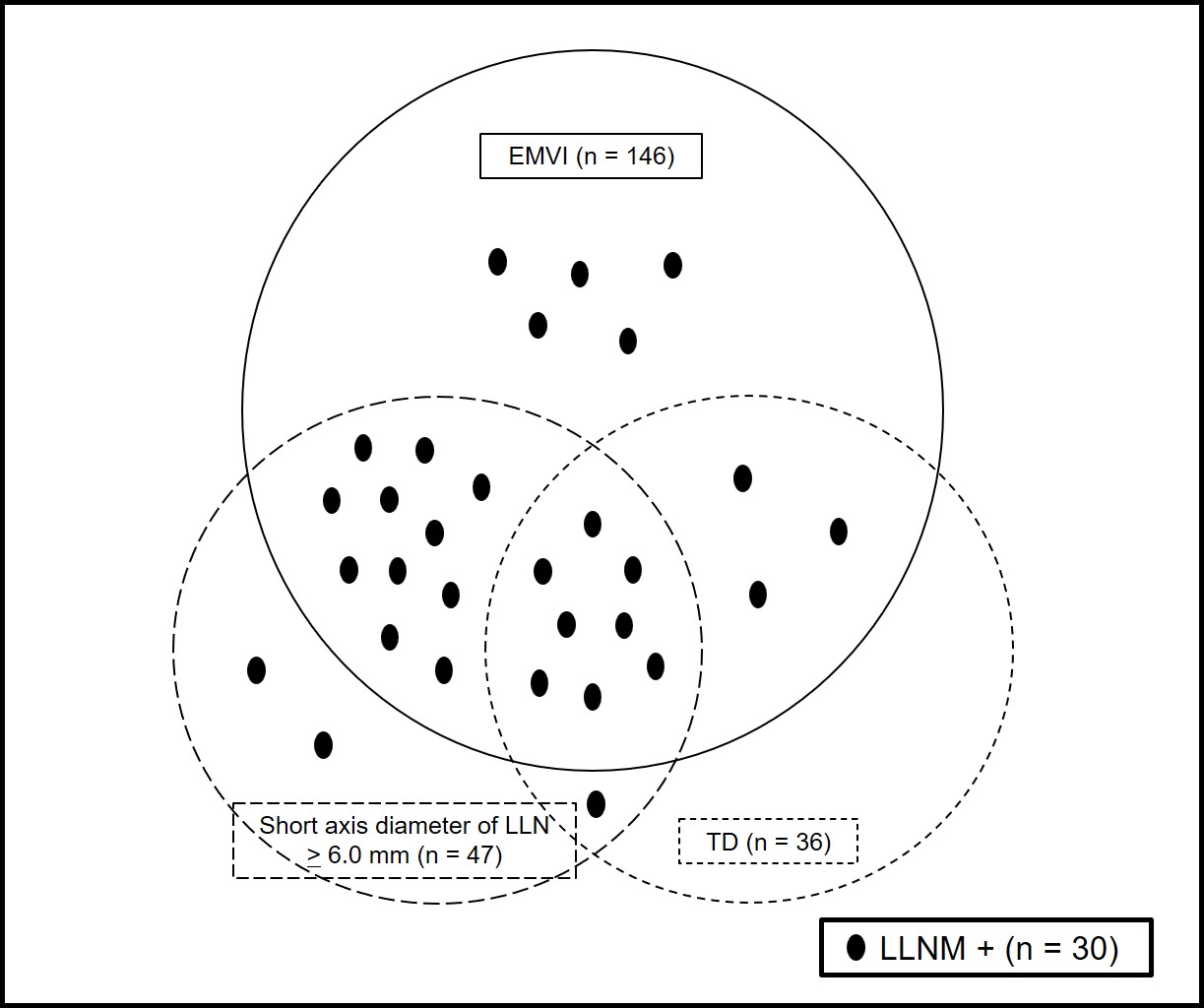


**Figure S2** Receiver operating characteristic curves of short- and long-axis diameter of lateral lymph node for diagnosing lateral lymph node metastasis

Since the cutoff values that maximized the sum of sensitivity and specificity were 6.6 mm and 8.9 mm for the short- and long-axis diameters of LLN, respectively, the cutoff values for diagnosing LLNM in the present study were set to 6.0 mm and 8.0 mm.

ROC, receiver operating characteristic; LLN, lateral lymph node; LLNM, lateral lymph node metastasis


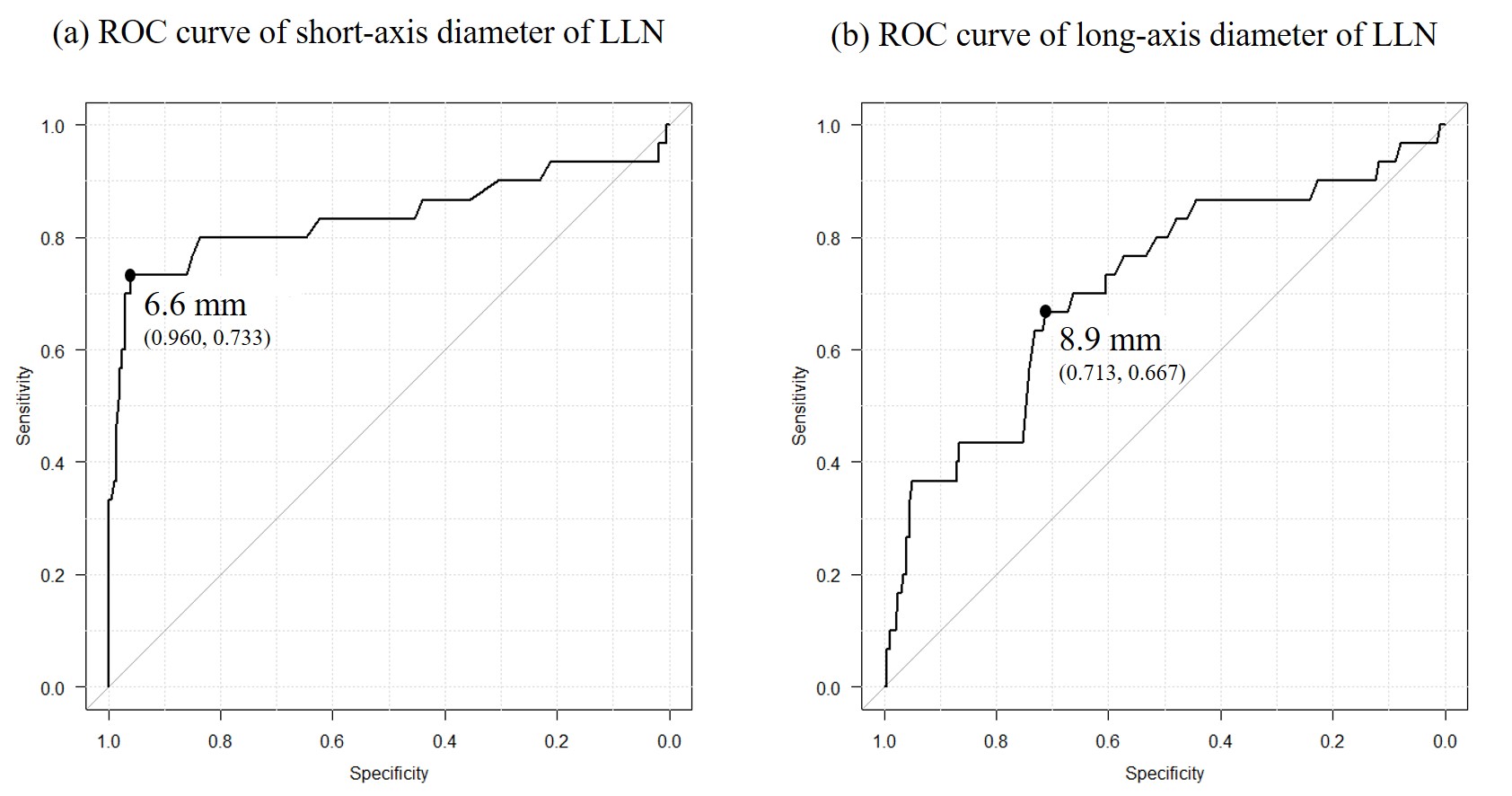


**Figure S3** SHapley Addictive exPlanations values of the support vector machine

SHAP, SHapley Addictive exPlanations; CEA, carcinoembryonic antigen; por, poorly differentiated adenocarcinoma; muc, mucinous adenocarcinoma; EMVI, extramural vascular invasion; TD, tumour deposit; and LLN, lateral lymph node


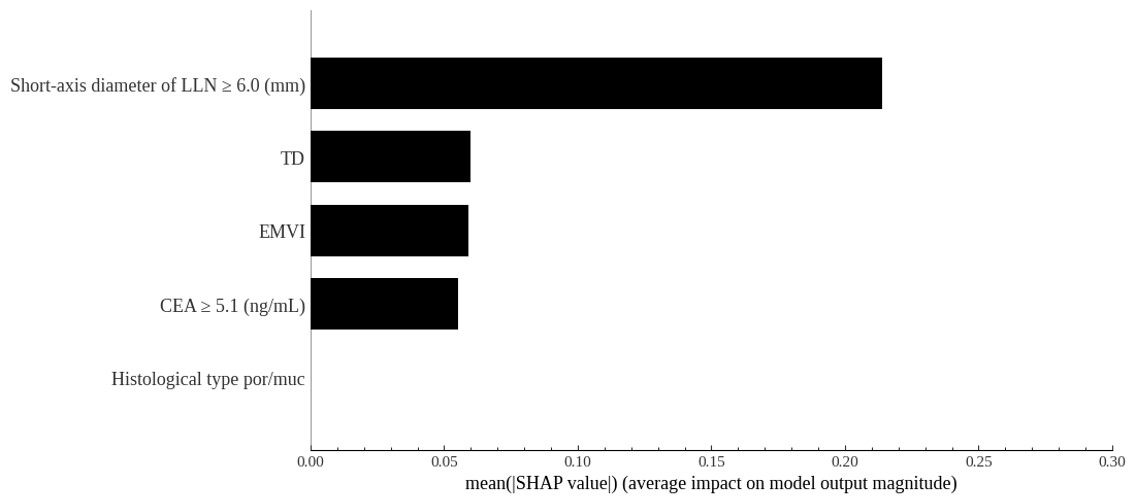


**Figure S4** SHapley Addictive exPlanations values of the light gradient boosting machine

SHAP, SHapley Addictive exPlanations; CEA, carcinoembryonic antigen; por, poorly differentiated adenocarcinoma; muc, mucinous adenocarcinoma; EMVI, extramural vascular invasion; TD, tumour deposit; and LLN, lateral lymph node


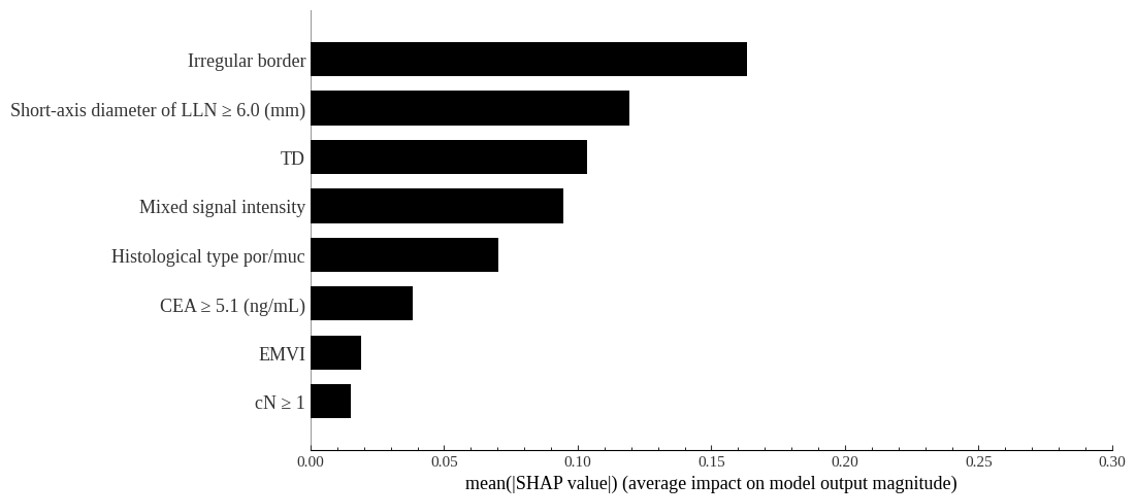


**Figure S5** SHapley Addictive exPlanations values of ensemble learning

SHAP, SHapley Addictive exPlanations; CEA, carcinoembryonic antigen; por, poorly differentiated adenocarcinoma; muc, mucinous adenocarcinoma; EMVI, extramural vascular invasion; TD, tumour deposit; and LLN, lateral lymph node


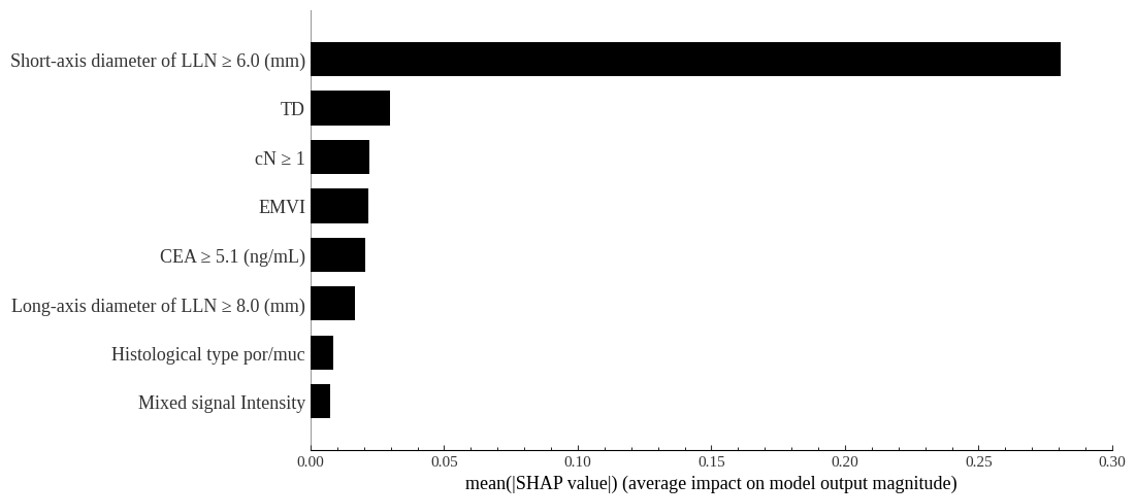


**Table S1** Range of hyperparameters explored in the optimisation using ‘Optuna’

| Algorithm | Hyperparameter | Minimum | Maximum |
| --- | --- | --- | --- |
| Logistic regression  (single/ensemble) | C | 1e-5 | 100 |
| LightGBM  (single/ensemble) | reg_alpha | 1e-3 | 10 |
|  | reg_lambda | 1e-4 | 0.1 |
|  | num_leaves | 2 | 6 |
|  | colsample_bytree | 0.4 | 1.0 |
|  | Subsample | 0.4 | 1.0 |
|  | subsample_freq | 0 | 7 |
|  | min_child_samples | 0 | 30 |
| XGBoost  (single/ensemble) | Subsample | 0.1 | 1.0 |
|  | colsample_bytree | 0.2 | 1.0 |
|  | reg_alpha | 1e-3 | 10 |
|  | reg_lambda | 1e-3 | 1.0 |
|  | learning_rate | 1e-3 | 1.0 |
|  | min_child_weight | 2 | 14 |
|  | max_depth | 1 | 8 |
|  | Gamma | 1e-3 | 1.0 |
| RF  (single/ensemble) | n_estimators | 10 | 300 |
|  | max_features | 'auto', 'sqrt', 'log2' | |
|  | max_depth | 1 | 1000 |
|  | criterion  (Ensemble only) | ‘gini’, ‘entropy’ | |
|  | ccp_alpha  (Single only) | 0 | 0.2 |
|  | min_samples_leaf  (Ensemble only) | 1 | 10 |
| SVM  (single/ensemble) | svm__gamma | 3e-2 | 100 |
|  | svm__C | 0.1 | 1000 |

LightGBM, light gradient boosting machine; XGBoost, extreme gradient boosting; RF, random forest; and SVM, support vector machine

**Table S2** Patient characteristics in the training and test cohorts

|  | Training cohort  (139 sides) | Test cohort  (93 sides) | p value |
| --- | --- | --- | --- |
| Age (years) | 63 [35–82] | 65 [39–82] | 0.873 |
| Sex |  |  |  |
| Male | 89 (64.0) | 60 (64.5) | 1.00 |
| Female | 50 (36.0) | 33 (35.5) |  |
| BMI (kg/m^2^) | 22.4 [16.3–32.9] | 22.5 [15.4–34.0] | 0.775 |
| CEA (ng/mL) | 4.0 [0.5–452.6] | 3.9 [0.6–452.6] | 0.643 |
| CA19-9 (U/mL) | 11.0 [2–545] | 14.0 [2–545] | 0.751 |
| Tumour distance from the anal verge (cm) | 5.0 [0–11.0] | 5.0 [0–10.0] | 0.777 |
| Histological type |  |  |  |
| papillary/ well differentiated/ moderately differentiated | 133 (95.7) | 87 (93.5) | 0.550 |
| poorly differentiated/ mucinous | 6 (4.3) | 6 (6.5) |  |
| cT |  |  |  |
| 1 | 0 (0.0) | 0 (0.0) | 0.915 |
| 2 | 10 (7.2) | 5 (5.4) |  |
| 3 | 103 (74.1) | 71 (76.3) |  |
| 4 | 26 (18.7) | 17 (18.3) |  |
| cN |  |  |  |
| 0 | 47 (33.8) | 26 (28.0) | 0.777 |
| 1 | 33 (23.7) | 26 (28.0) |  |
| 2 | 32 (23.0) | 21 (22.6) |  |
| 3 | 27 (19.4) | 20 (21.5) |  |
| cM |  |  |  |
| 0 | 126 (90.6) | 84 (90.3) | 1.00 |
| 1 | 13 (9.4) | 9 (9.7) |  |
| EMVI on MRI | 86 (61.9) | 60 (64.5) | 0.782 |
| TD on MRI | 21 (15.1) | 15 (16.1) | 0.855 |
| Irregular border on MRI | 27 (19.4) | 20 (21.5) | 0.740 |
| Mixed signal intensity on MRI | 23 (16.5) | 15 (16.1) | 1.00 |
| Long-axis diameter of LLN (mm) on MRI | 7.0 [1.8–28.2] | 7.8 [1.8–44.4] | 0.357 |
| Short-axis diameter of LLN (mm) on MRI | 4.0 [1.3–22.5] | 4.4 [1.8–13.7] | 0.363 |
| LLNM | 18 (12.9) | 12 (12.9) | 1.00 |

Values are indicated as frequencies (percentages) or medians [ranges].

BMI, body mass index; CEA, carcinoembryonic antigen; CA19-9, carbohydrate antigen 19-9; EMVI, extramural vascular invasion; MRI, magnetic resonance imaging; TD, tumour deposit; LLN, lateral lymph node; and LLNM, lateral lymph node metastasis
