# Supplementary figures and images for: Risk factors and development of machine learning diagnostic models for lateral lymph node metastasis in rectal cancer: multicentre study
Source: BJS Open. 2024 Jul 17;8(4):zrae073. doi: 10.1093/bjsopen/zrae073 (PMC11252850; doi:10.1093/bjsopen/zrae073)

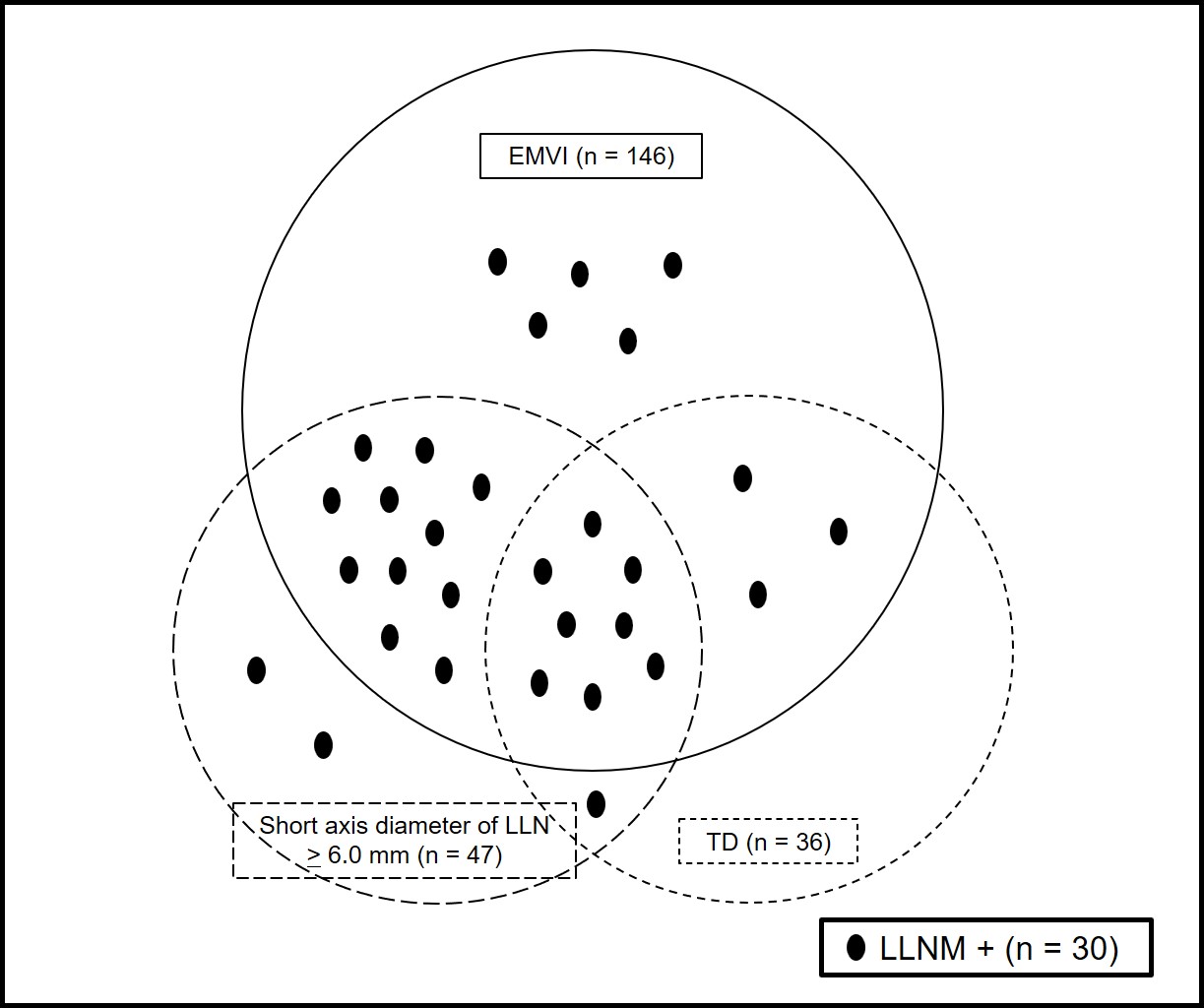

Supplement: zrae073_Supplementary_Data [file zrae073_supplementary_data.zip › FigureS1.jpg]

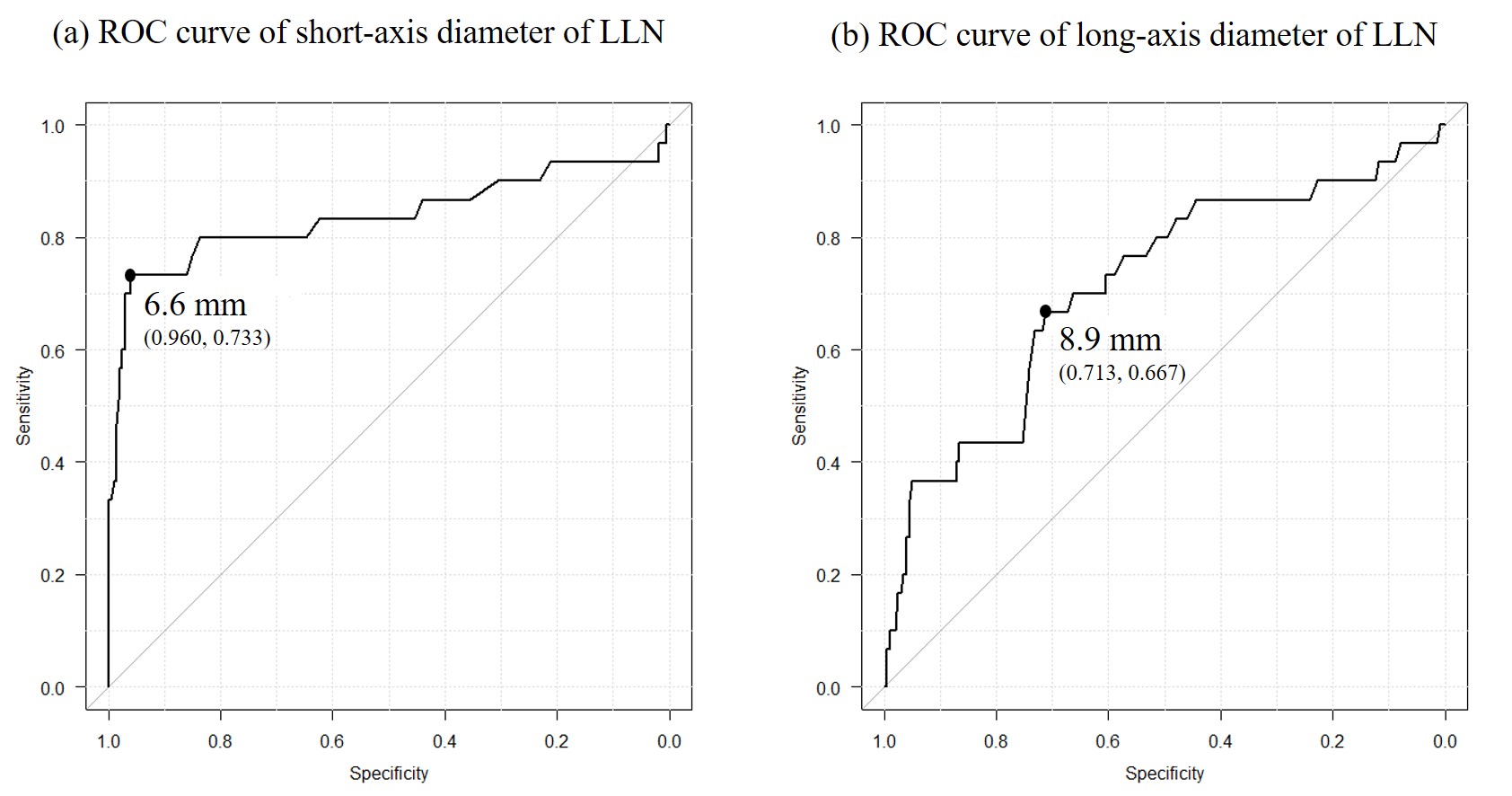

Supplement: zrae073_Supplementary_Data [file zrae073_supplementary_data.zip › FigureS2.jpg]

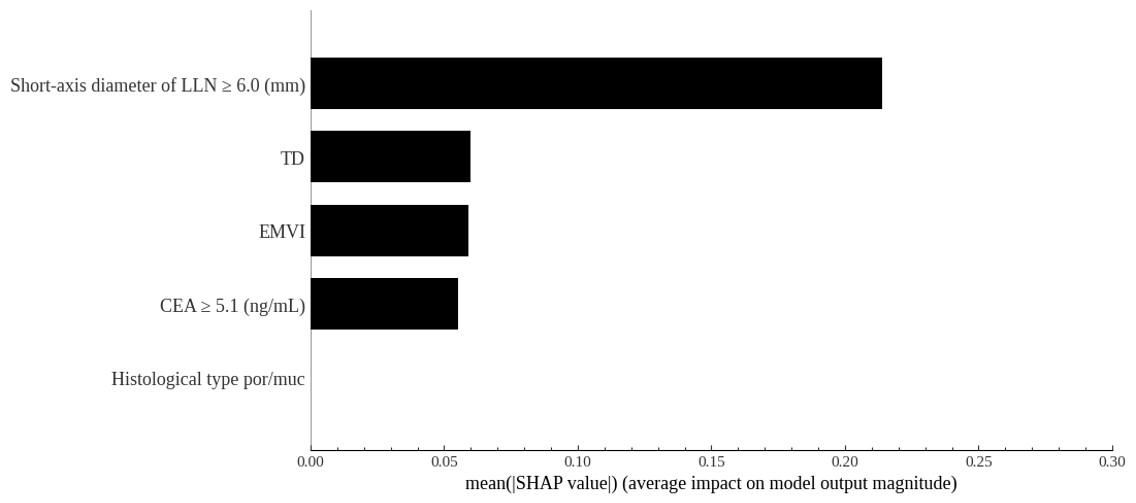

Supplement: zrae073_Supplementary_Data [file zrae073_supplementary_data.zip › FigureS3.jpg]

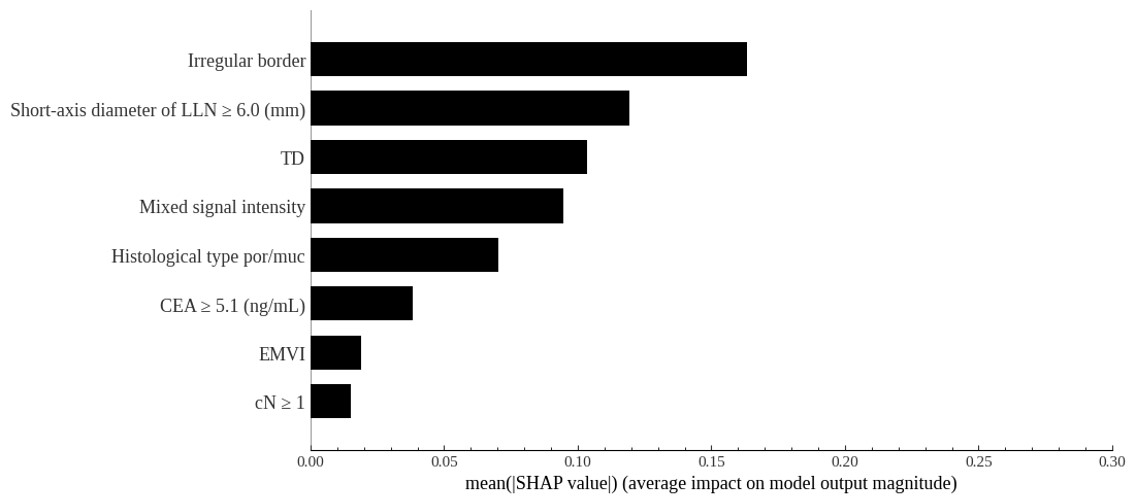

Supplement: zrae073_Supplementary_Data [file zrae073_supplementary_data.zip › FigureS4.jpg]

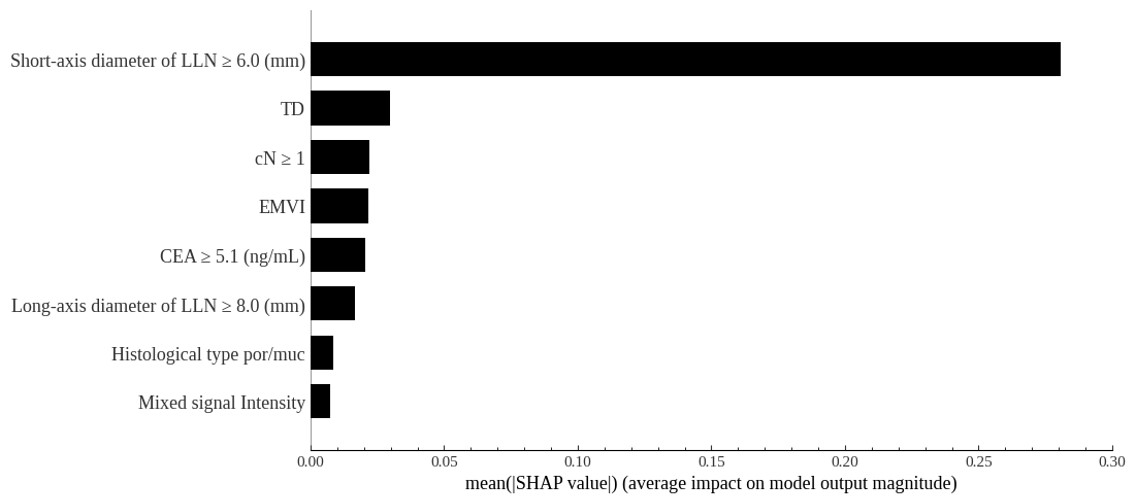

Supplement: zrae073_Supplementary_Data [file zrae073_supplementary_data.zip › FigureS5.jpg]
